# Roles of the Putative Type IV-like Secretion System Key Component VirD4 and PrsA in Pathogenesis of *Streptococcus suis* Type 2

**DOI:** 10.3389/fcimb.2016.00172

**Published:** 2016-12-02

**Authors:** Xiaowu Jiang, Yunkai Yang, Jingjing Zhou, Lexin Zhu, Yuanxing Gu, Xiaoyan Zhang, Xiaoliang Li, Weihuan Fang

**Affiliations:** Zhejiang University Institute of Preventive Veterinary Medicine, and Zhejiang Provincial Key Laboratory of Preventive Veterinary MedicineZhejiang, China

**Keywords:** *Streptococcus suis* type 2, streptococcal toxic shock syndrome, VirD4, anti- phagocytic, PrsA, inflammatory response

## Abstract

*Streptococcus suis* type 2 (SS2) is a zoonotic pathogen causing septic infection, meningitis and pneumonia in pigs and humans. SS2 may cause streptococcal toxic shock syndrome (STSS) probably due to excessive release of inflammatory cytokines. A previous study indicated that the *virD4* gene in the putative type IV-like secretion system (T4SS) within the 89K pathogenicity island specific for recent epidemic strains contributed to the development of STSS. However, the functional basis of VirD4 in STSS remains unclear. Here we show that deletion of *virD4* led to reduced virulence as shown by about 65% higher LD_50_, lower bacterial load in liver and brain, and lower level of expression of inflammatory cytokines in mice and cell lines than its parent strain. The ΔVirD4 mutant was more easily phagocytosed, suggesting its role as an anti-phagocytic factor. Oxidative stress that mimic bacterial exposure to respiratory burst of phagocytes upregulated expression of *virD4*. Proteomic analysis identified 10 secreted proteins of significant differences between the parent and mutant strains under oxidative stress, including PrsA, a peptidyl-prolyl isomerase. The SS2 PrsA expressed in *E. coli* caused a dose-dependent cell death and increased expression of proinflammatory IL-1β, IL-6 and TNF-α in murine macrophage cells. Our data provide novel insights into the contribution of the VirD4 factor to STSS pathogenesis, possibly via its anti-phagocytic activity, upregulation of its expression upon oxidative stress and its involvement in increased secretion of PrsA as a cell death inducer and proinflammatory effector.

## Introduction

*Streptococcus suis* type 2 (SS2) is a major swine pathogen and an emerging zoonotic agent, causing meningitis, arthritis, septicemia and pneumonia in humans and pigs (Lun et al., 2007; Gottschalk et al., 2010; Feng et al., 2014). *S. suis* type 2 was initially seen to cause only sporadic cases of meningitis and sepsis in humans working with pigs or pork-derived products (Wertheim et al., 2009). However, two outbreaks of human SS2 infection in China in 1998 and in 2005 raised considerable concerns among public health professionals (Tang et al., 2006). Infection in these two outbreaks was characterized by acute high fever, clear systemic erythematous blanching rash, disseminated intravascular coagulation and multiple organ failure and shock as well as short duration of the disease (acute death within hours of infection) and high mortality (Sriskandan and Slater, 2006; Tang et al., 2006).

*Streptococcus suis* type 2 isolates of these two outbreaks exhibit strong invasiveness and high pathogenicity leading to a new disease form as streptococcal toxic shock syndrome (STSS) which was originally referred to *S. pyogenes* (GAS) (Feng et al., 2010). The main virulence factors involved in *S. pyogenes* STSS consist of so-called superantigens or molecules that trigger non-specific, uncontrolled activation of T cells and massive cytokine release. Although a collection of new virulence factors have been described in SS2, there were no superantigen candidates identified so far, implying that a different mechanism could be involved in the STSS form caused by SS2 variant strains from these two outbreaks (Tang et al., 2006; Lappin and Ferguson, 2009; Feng et al., 2010). Comparative genomics analysis has revealed that a functional pathogenicity island (PAI) of about 89 kb (89K) is exclusively present in the epidemic strains in the two Chinese SS2 outbreaks but not in other clinical isolates (Chen et al., 2007; Feng et al., 2011). Further studies suggested that 89K PAI with a transposon-like essence could undergo GI-type (genomic island) T4SS (type IV secretion system)-mediated horizontal transfer and encodes at least two sets of genetic elements involved in SS2 virulence: a salK-salR two-component regulation system and a type IV-like secretion system (T4SS-like) (Zhao et al., 2011).

Another specific feature for STSS caused by SS2 variant isolates from the outbreaks was an early phase excessive burst of proinflammatory cytokines storm (Ye et al., 2009; Fittipaldi et al., 2012; Lachance et al., 2013). Such an unbalanced immune response could be harmful not only to the pathogens but also to the host. Such excessive inflammation is responsible for high mortality observed in STSS cases. A recent study illustrated that deletion of the key component (VirD4 or VirB4) of the T4SS-like system led to decreased virulence and alleviation of excessive systemic inflammatory responses in mice (Zhao et al., 2011). However, the functional mechanisms of the T4SS-like system (including VirD4) in SS2 infection are still poorly understood.

This study was aimed to investigate the roles of VirD4 in microbe-host interaction and to explore possible effectors contributing to the pathogenicity of *S. suis* type 2 infection.

## Materials and methods

### Bacterial strains and culture conditions

*Streptococcus suis* type 2 strain HA9801, kindly donated by Professor C. Lu (Nanjing Agricultural University, China), was an isolate from the Jiangsu outbreak of STSS in 1998. Its isogenic mutant with deletion of *virD4* ORF (ΔVirD4) was constructed using the pSET4s vector kindly provided by D. Takamatsu (National Institute of Animal Health, Japan) (Takamatsu et al., 2001). Deletion of the target gene was confirmed by PCR with flanking, internal and external primers (Figure S1). The SS2 strains were grown in Brain Heart Infusion (BHI) (Oxoid, England) at 37°C with shaking at 180 rpm. *Escherichia coli* DH5a and BL21 (TransGen, Beijing) were grown in Luria broth media (Oxoid, England).

### Virulence in murine model

A mouse infection model was used to compare virulence between ΔVirD4 mutant and its parent strain (or wild-type strain, WT). Bacterial cultures were adjusted to OD_600_ 0.4 with PBS (equivalent to about 3–5 × 10^8^ CFU/ml). 6-week old female BALB/c mice, eight for each strain, were infected by intraperitoneal injection of the two strains in 1.0 ml volume of the PBS dilutions containing 1.8 × 10^8^ to 1.2 × 10^9^ CFU. Mice inoculated with sterile PBS were included as control. Mortality was monitored every day for 7 days post-infection (dpi). The 50% lethal dose (LD_50_) was calculated using the Reed and Muench method. To compare bacterial load in organs, mice (six for each strain) were inoculated intraperitoneally with 4.3–4.5 × 10^8^ CFU. Mice were humanely euthanized at 12 h post-infection (hpi). Spleen, liver and brain samples were collected, homogenized and diluted for plate counting on BHI agar plates.

### Survival in murine blood

To examine the contribution of VirD4 in survival, Mouse whole blood survival assay was conducted as previously described (Liu et al., 2014). Briefly, 50 μl of the WT and ΔVirD4 strains at logarithmic phase adjusted to 0.4 in PBS were inoculated into sterile Eppendorf tubes pre-filled with 450 μl of fresh anticoagulant whole blood pooled from clinically healthy mice. The blood-bacteria mixtures were incubated with shaking at 180 rpm at 37°C for 1 h. Surviving bacteria were then diluted and plated on BHI agar plates for enumeration. Survival percentage was calculated as (CFU on plate/CFU in initial inoculum) × 100%.

### Phagocytosis assay

Phagocytosis assay was performed as previously described (Feng et al., 2012) in four types of cells, i.e., primary bone marrow macrophages (BMDM) from C57BL/6 mice (Hu et al., 2014), mouse macrophage cell lines RAW264.7, porcine alveolar macrophage 3D4/2 (PAM 3D4/2) and human monocytic cell line THP-1. Briefly, cells (2–2.5 × 10^5^ cells) grown in DMEM or 1640 medium (Gibco, Invitrogen, Germany) were infected at a multiplicity of infection (MOI) of 100 for 1 h. The infected monolayers were washed twice with warm PBS and re-incubated for another hour in the medium containing gentamicin (300 μg/ml) (Sigma-Aldrich, Ontario, Canada) to kill extracellular bacteria. Cultures were then washed four times with PBS and replaced with 1 ml of sterile distilled water to lyse cells. Viable intracellular bacteria were determined by plating of serial dilutions of the lysates on BHI agar. This assay was repeated at least four times with each strain tested in triplicate wells in independent experiments. Double immuno-fluorescence was performed to visualize phagocytosis as previously described (Benga et al., 2004). The RAW264.7 or BMDM cells were infected as above and washed three times with PBS. The cells were fixed for 10 min with 4% paraformaldehyde in PBS and blocked for 1 h with 10% inactivated fetal bovine serum in PBS. The macrophage cells were then sequentially probed with rabbit anti-*S. suis* antibody (1:100) for 1 h and TRITC-conjugated goat anti-rabbit IgG antiserum (1:50, Boster, China) for 1 h to stain extracellular bacteria. Afterwards, cells were washed with PBS and permeabilized with 0.2% Triton X-100 for 15 min. Total cell-associated bacteria were identified by staining of permeabilized cells with the same primary antibody followed by FITC-conjugated goat anti-rabbit IgG antibody for 1 h. Samples were analyzed with 600-fold magnification on an IX-81 confocal microscope integrated into the FV-1000 imaging system (Olympus, Hamburg, Germany).

### Analysis of proinflammatory cytokines

BALB/c mice (four mice per strain at each time point) were inoculated intraperitoneally with sublethal dose (2.1–3.3 × 10^8^ CFU) of WT and ΔVirD4 strains. Mice injected with sterile PBS were included as control. Spleen, brain and blood samples were collected at 6, 12, and 24 hpi. Total RNA samples from spleens and brains were isolated using animal tissue total RNA isolation kit (TIANGEN, Beijing) and used for cDNA synthesis with AMV reverse transcriptase reaction kit (TOYOBO, Japan) according to the manufacturer's instructions. Quantitative PCR (qPCR) was performed to measure the transcriptional levels of proinflammatory cytokines (IL-β, IL-6, TNF-α and MCP-1) using the SYBR green PCR Kit (TOYOBO) and the Agligent MX3000P qPCR system (Stratagene, USA). Additionally, macrophage cell lines RAW264.7 and 3D4/2 were infected at MOI of 100 for 2 h. Total RNA isolation, cDNA synthesis and qPCR were conducted as described above. All primers used for qPCR were listed in Table S1. β*-actin* was used as the reference gene for normalization. Results were presented as fold changes relative to non-infected cells using the 2^−ΔΔCt^ method. IL-1β and IL-6 cytokines in serum samples were determination by ELISA kits (ExCell, Shanghai).

### Analysis of secreted protein profiles and Vird4 expression of *S. suis* type 2 exposed to hydrogen peroxide stress or in infected macrophages

Pathogenic bacteria are faced with oxidative stress during infection as a result of respiratory burst of the phagocytic cells. To mimic such oxidative stress, the WT and ΔVirD4 strains were cultured at 37°C in BHI with or without 10 mM H_2_O_2_. Bacterial cells were collected at late logarithmic phase. Total bacterial RNA was extracted for cDNA synthesis with the reverse transcriptase reaction kit (TOYOBO). qPCR was performed to measure the mRNA level of the *virD4* gene using specific primers (Table S1). To examine the role of cellular effects in *virD4* expression, RAW264.7 cells were infected with the wild-type strain (MOI 100 for 2 h) in the presence or absence of a reactive oxygen species inhibitor (N-acetyl-l-cystein, NAC, 5 mM). Two parts of the bacterial population were collected for qPCR analysis of *virD4* mRNA: bacteria adhered to and phagocytosed by the macrophages (cell-associated) and unassociated supernatant bacteria (bacteria in culture supernatant). To analyze possible involvement of VirD4 in protein secretion upon H_2_O_2_ treatment, culture supernatant samples of both the WT and ΔVirD4 strains were prepared as previously reported (Geng et al., 2008). Briefly, culture supernatants at late logarithmic phase BHI cultures with or without H_2_O_2_ treatment were harvested by centrifugation (8000 rpm for 20 min at 4°C), treated with a protease inhibitor PMSF (Phenylmethanesulfonyl fluoride) (Beyotime, Shanghai) and filtered through a 0.22-μm membrane. The secreted proteins were precipitated with 10% trichloroacetic acid (TCA) in acetone for 1 h on ice and collected by centrifugation at 10,000 × g for 20 min at 4°C. The precipitates were washed 3 times with ice-cold acetone containing 0.1% PMSF and air-dried. Proteins were quantified with the BCA kit (Beyotime, Shanghai). The protein samples (30 μg per lane) were subjected to SDS-PAGE followed by Coomassie blue R250 staining or by Western blotting using rabbit anti-SS2 whole cell polyclonal antibodies (1:300) and HRP-conjugated goat anti-rabbit secondary antibodies. Protein bands were visualized using the chemi-luminescence substrate (Thermo, USA) and chemiluminescent imaging system (SageCreation, Beijing).

### Identification of secreted proteins by 2D-page and LC-MS/MS

The above protein samples were further treated with a 2D Clean-Up kit (Bio-Rad), resuspended in lysis buffer and then quantified for equal loading. Two dimensional electrophoresis (2DE) and identification of proteins by mass spectrometry (Bruker Dalton, Ultraflex III TOF/TOF) were performed as described elsewhere (Jing et al., 2008). Comparison of secreted protein spots was performed with the ImageMaster 2D platinum 5.0 (GE Healthcare, Uppsala, Sweden). Only spots with relative high abundance showing consistent and reproducible changes (>3 folds) in 2D-gels were excised and subjected to mass spectrometry. FlexAnalysis software (Bruker Dalton) was used to remove contaminant peaks (including matrix peaks and solvent peaks). Peptide mass fingerprinting (PMF) data was analyzed using the MASCOT server (http://www.matrixscience.com) to search for target proteins from the *S. suis* protein sequence data in the NCBI database.

### Transcriptional analysis of the genes encoding differentially secreted proteins upon oxidative stress

Procedures for oxidative stress, total RNA extraction from the WT and ΔVirD4 strains and cDNA synthesis were described above. qPCR was conducted using specific primers (Table S1) to evaluate transcriptional levels of the genes encoding significantly upregulated or downregulated proteins between WT and ΔVirD4 strains. The *16S rRNA* gene was tested in parallel for normalization.

### Expression of recombinant PrsA protein and antiserum preparation

The PrsA gene was PCR-amplified from the genome DNA of the strain HA9801 using specific primers (Table S1), digested with corresponding restriction enzymes, and cloned to pET-30a vector for expression in *E. coli* BL21. The recombinant protein was purified using Ni-NTA columns (Novagen). Lipopolysaccharides in the purified protein were removed using a ToxinEraser™ Endotoxin Removal Kit (Genscript, USA), and tested using a Chromogenic LAL Endotoxin Assay Kit (GenScript, USA). The protein was then passed through a 0.22-μm filter, concentrated by membrane ultrafiltration (Millipore) and stored at −80°C. The BCA protein assay kit (Beyotime) was used to determine the protein concentration. SDS-PAGE and Western blotting were performed to confirm presence of the target protein with an anti-His monoclonal antibody (Sungene Biotech, China). Hyperimmune sera to PrsA were obtained from New Zealand White rabbits after four times of subcutaneous immunization at a 2-week interval with 200 μg of purified recombinant PrsA emulsified with Freund's complete (primary immunization) or incomplete adjuvant (booster immunization). Anti-PrsA sera were collected and had titers >1:10000 as measured by ELISA. Preimmune serum samples were collected as negative control. In order to measure the relative abundance of PrsA in the culture supernatants of the WT and ΔVirD4 strains treated with or without hydrogen peroxide, the extracted protein samples (3 μg/well) were coated onto the 96-wells and probed with rabbit anti-PrsA polyclonal antibodies for indirect ELISA.

### Investigation of PrsA protein functions

Cell cytotoxicity of PrsA was conducted as previously described (Jiang et al., 2016). The mouse brain microvascular endothelial cell line bEND3.0 cultured in DMEM supplemented with 10% FBS (Gibco, USA) were stimulated with purified PrsA at different concentrations and incubated for 1 or 2 h at 37°C with 5% CO_2_. PBS and 0.2% Triton X-100 treatments served as negative and positive controls, respectively. Cytotoxicity was qualified by Lactate Dehydrogenase Cytotoxicity Assay Kit (Beyotime, China) or observed directly under fluorescent microscope (×100) after staining with Live/Dead cytotoxic kit (Invitrogen, Eugene, OR).

Hemolysis assay was conducted as elsewhere described (Zheng et al., 2011). Purified PrsA protein was added to 2% sheep red blood cells in PBS at final concentration of 50 and 100 μg per ml. The mixtures were incubated for 1 h at 37°C with 5% CO_2_. After brief centrifugation at 800 g for 10 min, supernatant samples were measured at OD_540_ on the SpectraMax^M2^ Microplate reader (Molecular Devices, USA). Erythrocytes with His-tag, bovine serum albumin or 0.2% Triton-100 and those without any treatment served as controls.

To analyze whether PrsA could induce release of pro-inflammatory cytokines, RAW264.7 cells (2–2.5 × 10^5^ cells) were seeded into 24-well cell culture plates (Corning, USA) and incubated in DMEM with 10 μg/ml of PrsA or 400 ng/ml LPS (positive control) and without treatment (DMEM only as negative control) at 37°C with 5% CO_2_. Culture supernatants were collected at indicated times for determination of IL-1β and TNF-α using commercially available ELISA Max Deluxe kits (Biolegend, USA). These two cytokines and IL-6 were also analyzed at the transcriptional level by qPCR as described above.

### Animal ethics

All animal experiments were conducted following the International Guiding Principles for Biomedical Research Involving Animals-1985 and approved protocols of the Laboratory Animal Management Committee of Zhejiang University (Approval No. 2015016).

### Statistical analysis

All experiments were performed in triplicate and repeated at least three times. Experimental results were expressed as mean ± SD unless otherwise stated. Data were subjected to normality tests and analyzed using two-tailed Student's *t*-test with *P* values < 0.05 or 0.01 considered as significant.

## Results

### VirD4 played a role in virulence of *S. suis* type 2

**Figure 1:**
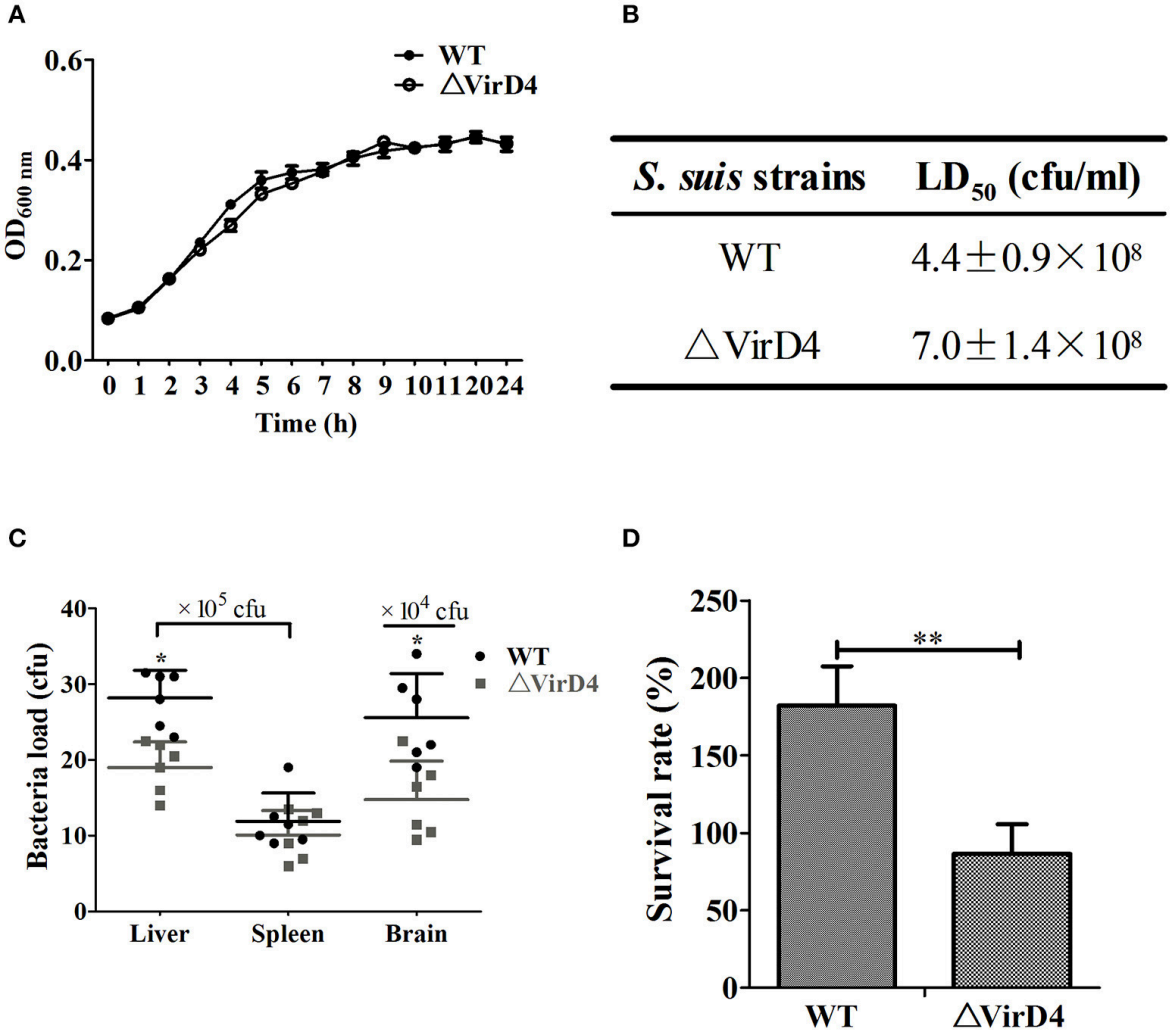
**The ΔVirD4 mutant was less virulent than the wild-type strain. (A)** Growth curves of the wild-type (WT) *S. suis* type 2 strain HA9801 and ΔVirD4 mutant. Both strains were cultured in BHI broth and optical density was measured over a 12-h period. **(B)** 50% lethal dose of WT and ΔVirD4 strains. Different levels of bacteria were intraperitoneally inoculated into BALB/c mice and observed for mortality over a 1-week period. **(C)** Bacterial load in mice organs (*n* = 6) at 12 h after sublethal intraperitoneal infection with WT and ΔVirD4 strains. Each point represents a single sample. **(D)** Survival of WT and ΔVirD4 strains in mice blood for 1 h. Data were shown as mean ± SD of three repeated experiments, each in triplicate tubes, ^*^ at *P* < 0.05 or ^**^P < 0.01.

To determine the role of VirD4 in virulence, an isogenic mutant with deletion of *virD4*-ORF (ΔVirD4) was constructed from the wild-type strain HA9801 (Figure S1). Deletion of *virD4* did not affect growth in BHI broth with or without hydrogen peroxide treatment, as compared with its parent strain (Figure 1A and Figure S2). With BALB/c mouse infection model, we found that VirD4 contributed to virulence as shown by about 65% higher LD_50_ (Figure 1B) and lower bacterial load in liver and brain (*P* < 0.05; Figure 1C) than its parent strain. The ΔVirD4 mutant was also more susceptible to bactericidal effect of the whole blood than the wild-type strain (*P* < 0.01, Figure 1D).

### VirD4 was anti-phagocytic

**Figure 2:**
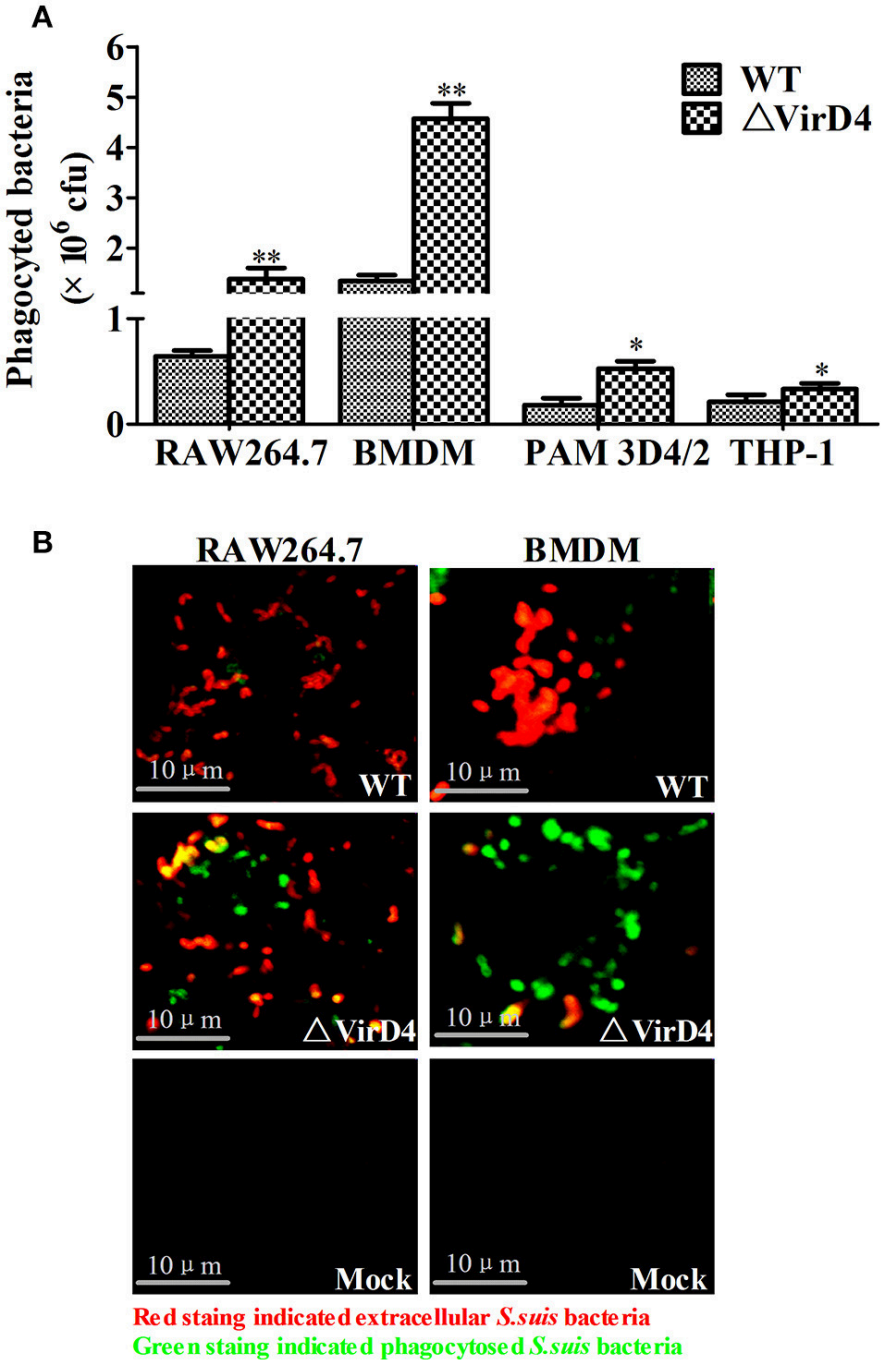
**Deletion of *virD4* rendered the bacteria more easily phagocytosed by murine or porcine macrophage cell lines. (A)** Primary bone marrow macrophages (BMDM) from C57BL/6 mice and murine macrophage cell line RAW264.7, porcine alveolar macrophage (PAM) 3D4/2 and human monocytic cell line THP-1 were infected with wild-type (WT) *S. suis* type 2 or ΔVirD4 mutant strains (MOI at 100) for 2 h, respectively. After 1 h treatment with gentamicin, phagocytosed bacteria were counted on BHI agar plates. All experiments were repeated at least three times (each in triplicate samples) and showed similar trends. Data were shown as mean ± SD of three replicate wells of a typical experiment. “^*^” or “^**^” indicated significant at *P* < 0.05 or *P* < 0.01, respectively. **(B)** Immunofluorescence for observation of phagocytosed bacteria. Bacteria were stained by rabbit anti-SS2 whole cell serum. Extracellular and intracellular bacteria were distinguished by red (TRITC) and green (FITC)-colored secondary antibody staining. Bar scale indicated were 10 μm.

Phagocytic cells serve as the first line of host defense against invading bacterial pathogens. We investigated whether VirD4 could play roles in phagocytosis using murine BMDM and RAW264.7, porcine PAM 3D4/2 cells as well as human derived THP-1 cells. Surprisingly, deletion of *virD4* rendered the bacteria more easily phagocytosed, particularly in BMDM (Figure 2A). Double label immuno-fluorescence assay further verified this phenomenon (Figure 2B). These findings indicated that VirD4 might be an anti-phagocytic factor.

### Deletion of *VirD4* decreased expression of inflammatory cytokines

**Figure 3:**
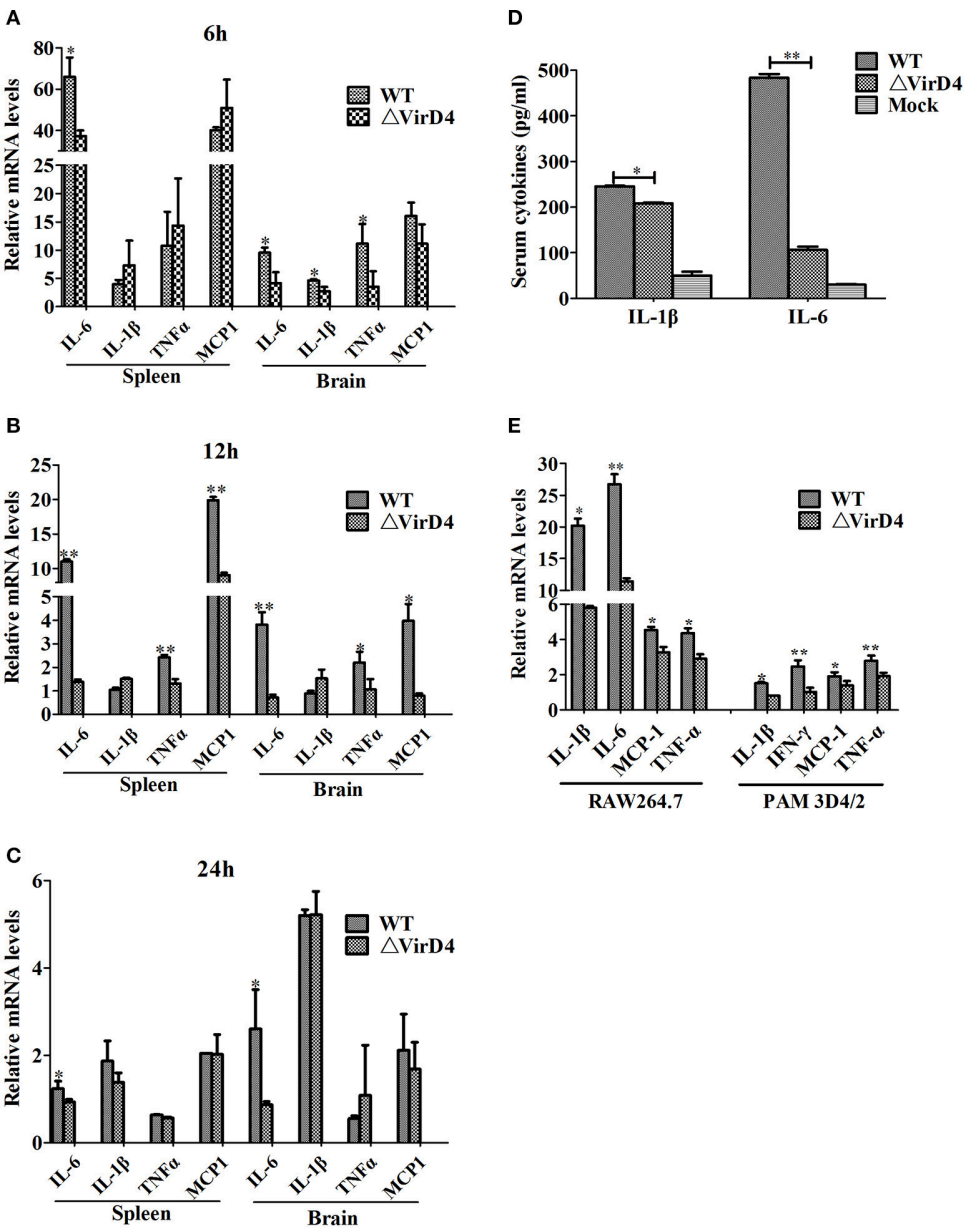
**Deletion of *virD4* led to reduced proinflammatory responses in *S. suis* type 2 infected mice or cultured cells. (A–C)** Transcriptional analysis of selected proinflammatory cytokines in spleen and brain from BALB/c mice infected with infected with a sublethal dosage of wild-type (WT) *S. suis* type 2 or ΔVirD4 mutant strains at 6, 12, and 24 hs (*n* = 4 per strain at each time point). **(D)** IL-1β and IL-6 levels in serum samples of BALB/c mice (*n* = 4 per strain) at 6 hs after infection measured by ELISA. **(E)** Relative transcriptional levels of inflammatory associated cytokines in murine or porcine cell lines by qPCR. The results are presented as fold induction relative to non-infected cells against β*-actin* gene expression using 2^−ΔΔCt^ method. Murine RAW264.7 or porcine PAM 3D4/2 macrophage cells were stimulated by wild-type (WT) *S. suis* type or ΔVirD4 mutant strains (MOI = 100) for 2 hs, respectively. Data were shown as mean ± SD of three experiments. “^*^” or “^**^” indicated significant at *P* < 0.05 or *P* < 0.01, respectively. MCP-1, monocyte chemotactic protein-1.

To determine whether VirD4 is involved in expression of proinflammatory cytokines, serum or organ samples were collected from intraperitoneally infected mice for analysis of proinflammatory cytokines. Figure 3 showed that *S. suis* could induce a rapid proinflammatory systemic response in mice with systemic cytokine and chemokine release. The transcriptional levels of IL-6, TNF-a, IL-1β and MCP-1 were remarkably increased in the early stage of infection (within 12 h) and gradually decreased to their basal levels by 24 hpi (Figures 3A–C). IL-6, TNF-a, and MCP-1 levels induced by WT strain were obviously higher than ΔVirD4 strain infection at 12 hpi. Because the bacterial load of the WT strain was higher than that of the virD4 mutant in the mice brain (Figure 1C), the observed differences in mRNA transcripts of cytokines in mice brain could be attributed partly to bacterial load. Serum levels of IL-6 and IL-β in mice infected with ΔVirD4 strain at 6 hpi were significantly lower than those infected with the WT strain (Figure 3D). Proinflammatory cytokines from RAW264.7 and PAM3D4/2 macrophage cells infected with ΔVirD4 strain were also lower than the WT strain at the transcriptional level, specifically for IL-1β and IL-6 (Figure 3E).

### Oxidative stress activated VirD4 factor and deletion of *virD4* changed profiles of secreted proteins

**Figure 4:**
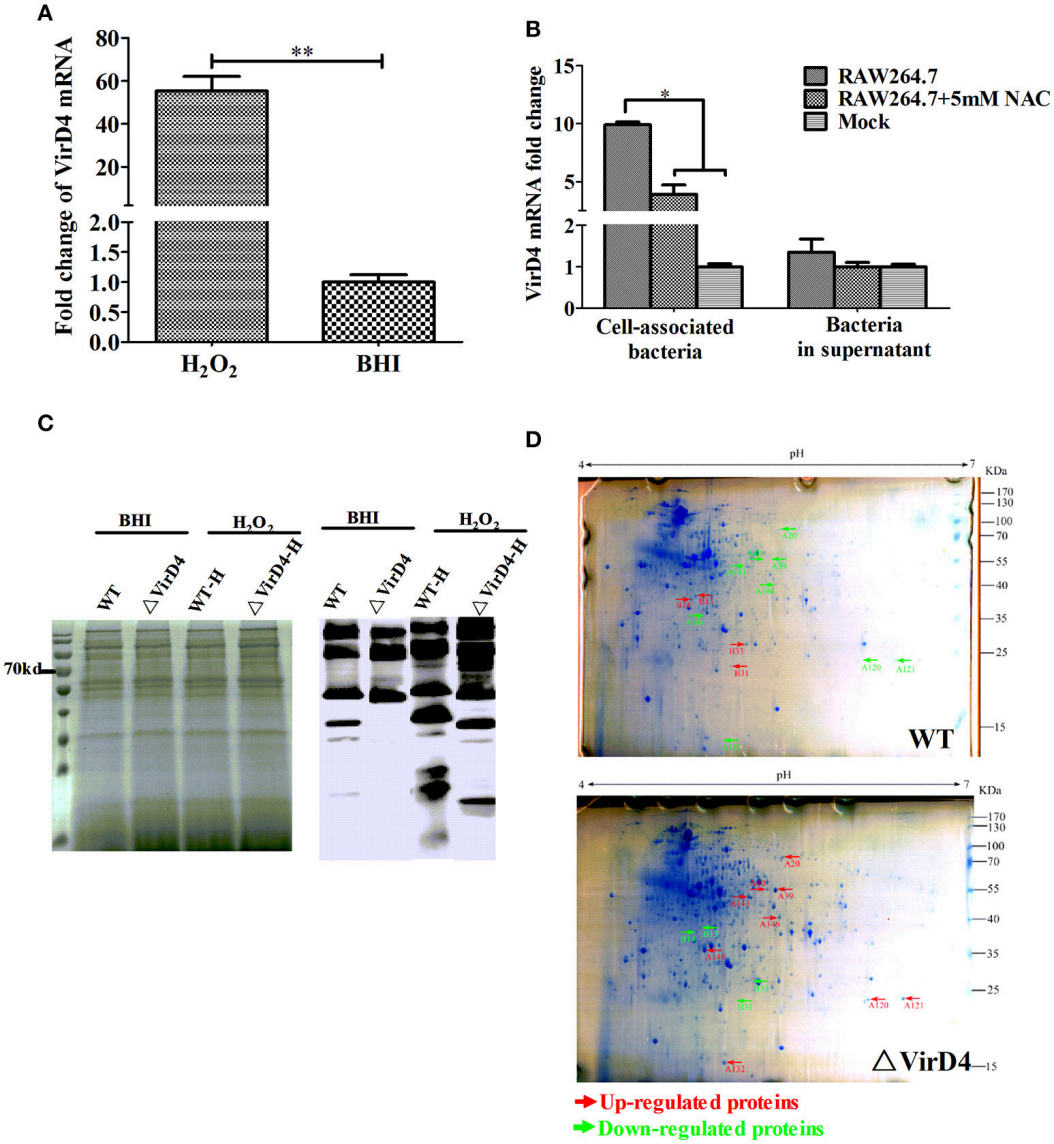
**The *virD4* gene was induced by hydrogen peroxide treatment and its deletion could induce differentially secreted protein profiles between wild-type (WT) *S. suis* type 2 and ΔVirD4 strains subjected to oxidative stress. (A)** The *virD4* mRNA level from the wild-type *S. suis* type 2 strain subjected to hydrogen peroxide treatment. ^**^Indicated significant at *P* < 0.01. **(B)** The *virD4* mRNA transcripts of the cell-associated bacteria and unassociated supernatant bacteria (bacteria in supernatant) after 2 hs infection in RAW264.7 cells in the presence or absence of N-acetyl-l-cystein (NAC), a reactive oxygen species inhibitor. ^*^Indicated significant at *P* < 0.05. **(C)** Differentially secreted proteins were found in regular SDS-PAGE gels and confirmed by Western blotting. **(D)** Differentially secreted supernatant protein profiles detected by 2DE. Red and Green arrows indicate some upregulated or down-regulated proteins in the wide-type or ΔVirD4 strains compared between the two gels that were used for LC/MS.

Early studies identified some *S. suis* type 2 proteins induced *in vivo*, including the T4SS-like VirD4 component using the *in vivo*-induced antigen technology (IVIAT) (Gu et al., 2009; Li et al., 2013). One of the major *in vivo* factors that the invading bacteria encounter is reactive oxygen species (ROS, including hydrogen peroxide) resulting from respiratory burst (Forman and Torres, 2002; Fittipaldi et al., 2012). We attempted to mimic *in vivo* environment by exposing the WT and ΔVirD4 strains to H_2_O_2_ added to the BHI medium at 10 mM with a sub-inhibitory effect on growth in the pilot test (Figure S2). The *virD4* mRNA level was about 50 folds higher after H_2_O_2_ stress (Figure 4A, *P* < 0.001), indicating that VirD4 could be an *in vivo* induced gene. Figure 4B shows that virD4 mRNA in cell-associated bacteria was significantly higher than that of mock control (*P* < 0.05), a finding similar to oxidative stress assay (Figure 4A). Treatment of the cells with ROS inhibitor (N-acetyl-l-cystein, NAC at 5 mM) reduced virD4 expression (*P* < 0.05). Thus, we believe that up-regulation of virD4 mRNA is mainly due to oxidative stress. There were also significant differences of secreted proteins between WT and ΔVirD4 strains exposed to H_2_O_2_ as shown by Western blot and SDS-PAGE (Figure 4C). These findings prompted us to use two dimensional gel electrophoresis to separate the supernatant proteins of WT and ΔVirD4 strains exposure to H_2_O_2_. The differentially expressed proteins with high abundance were marked in 2DE gels for further identification by mass spectrometry (Figure 4D).

### Confirmation of the selected differentially secreted proteins by qPCR

Table 1 showed that 148 protein spots were upregulated in the ΔVirD4 strain exposed to hydrogen peroxide stress while only 33 protein spots with 1.5-fold or higher upregulation in the WT strain. Mass spectrometry was used to identify protein spots of significant upregulation: four spots were identified as parvulin-like peptidyl-prolyl isomerase (PrsA), succinate dehydrogenase (SucD), and glycerate-dependent phosphoglycerate mutase (GpmA) in the WT strain with spots B13 and B14 representing isoforms of the same protein PrsA (Table 2). Nine spots were determined in the ΔVirD4 strain as ribonucleoside-diphosphate reductase (RNR), 6-phospho-beta-galactosidase (LacG), uracil phosphoribosyltransferase (UPP), galactose-6-phosphate isomerase subunit (LacB), arginine deiminase (arcA), ornithine carbamoyltransferase (arcB) and tagatose-bisphosphate aldolase (LacD). The spots A39 and A40 or those of A120 and A121 represent isoforms of LacG or LacD, respectively. These proteins were mainly involved in DNA damage repair, nucleotide biosynthesis, carbohydrate metabolism and stress modulation. These up-regulated proteins were confirmed by qPCR (Table 2).

**Table 1:** **Summarization of differentially secreted proteins between wild-type and ΔVirD4 strains separated on the two-dimensional PAGE gels**.

**Cells**	**No. of protein spots showing changes**	**1.5- to 3-fold changes**	**3- to 5-fold changes**	**>5 fold changes**	>10000^*^ fold change
WT	33	14	5	3	11
ΔVirD4	148	91	29	12	16

* *indicates presence of the protein spots only in the wild-type or ΔVirD4 strain*.

**Table 2:** **Identification and qualification of differentially secreted proteins between wild-type and ΔVirD4 strains selected for LC/MS and qPCR**.

**Spots^a^**	**Proteins identified**	**Score**	**% coverage**	**Matched Peptides**	**Locus tag (05ZYH33)**	**Protein change in abundance^b^**	**Change in mRNA level**
B13	Parvulin-like peptidyl-prolyl isomerase (PrsA)	102	18	6	SSU05_1238	8.8	1.5
B14		115	21	7		3.8	
B31	Succinate dehydrogenase/fumarate reductase (SucD)	198	32	8	SSU05_2153	10000	1.7
B33	Glycerate-dependent phosphoglycerate mutase (GpmA)	295	33	8	SSU05_1638	9.7	1.4
A20	Ribonucleoside-diphosphate reductase (RNR)	541	32	25	SSU05_1207	7.8	27
A39	6-phospho-beta-galactosidase (LacG)	464	27	13	SSU05_1036	10000	30
A120	Uracil phosphoribosyltransferase (UPP)	354	57	14	SSU05_1553	12.2	13
A132	Galactose-6-phosphate isomerase subunit (LacB)	218	50	10	SSU05_1042	10000	68
A143	Arginine deiminase (arcA)	514	50	26	SSU05_0624	3.7	1.6
A146	Ornithine carbamoyltransferase (arcB)	348	56	24	SSU05_0626	5.0	2.2
A148	Tagatose-bisphosphate aldolase (LacD)	422	49	12	SSU05_1040	10000	102

a *B or A means these spot numbers were from differential expressed proteins in the wild-type strain or ΔVirD4 mutant, respectively as shown in Figure 4C*.

b *The number “10000” means that these proteins were present only in wild-type or ΔirD4 strain*.

### PrsA induced epithelial cells death and was a novel proinflammatory factor in *S. suis* type 2

**Figure 5:**
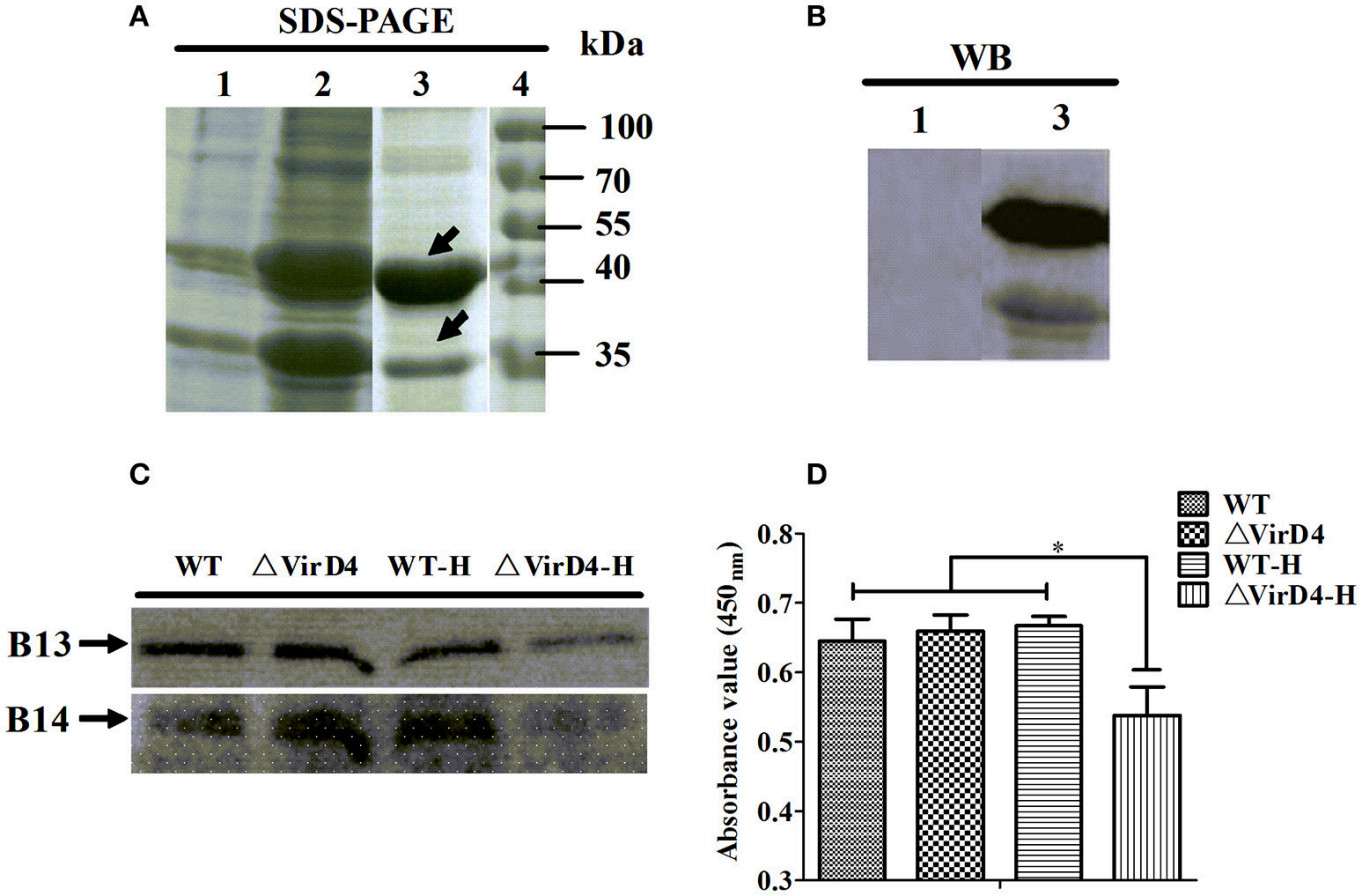
**Verification of more secretion of PrsA protein in wide-type strain as compared with its ΔVirD4 mutant after hydrogen peroxide treatment. (A)** Prokaryotic expression and purification of PrsA protein by SDS-PAGE. Lane 1, *E. coli* cell lysate from culture without IPTG induction; Lane 2, *E. coli* cell lysate from IPTG-induced culture; Lane 3, purified PrsA proteins, Lane 4, molecular weight markers. **(B)** Western blotting of Lane 1 and Lane 3 of **(A)** using anti-His monoclonal antibody. **(C,D)** Secretion of PrsA protein in the culture supernatant samples of the wild-type *S. suis* type 2 strain and its ΔVirD4 mutant after hydrogen peroxide treatment by Western blot **(C)** and ELISA **(D)** using the rabbit anti-PrsA polyclonal antibodies for probing. WT-H or ΔVirD4-H means the wild-type or ΔVirD4 strain were treated with 10 mM H_2_O_2_. ^*^Indicated significant at *P* < 0.05. The arrows in **(A)** indicate two possible isoforms of PrsA [same for **(C)**].

**Figure 6:**
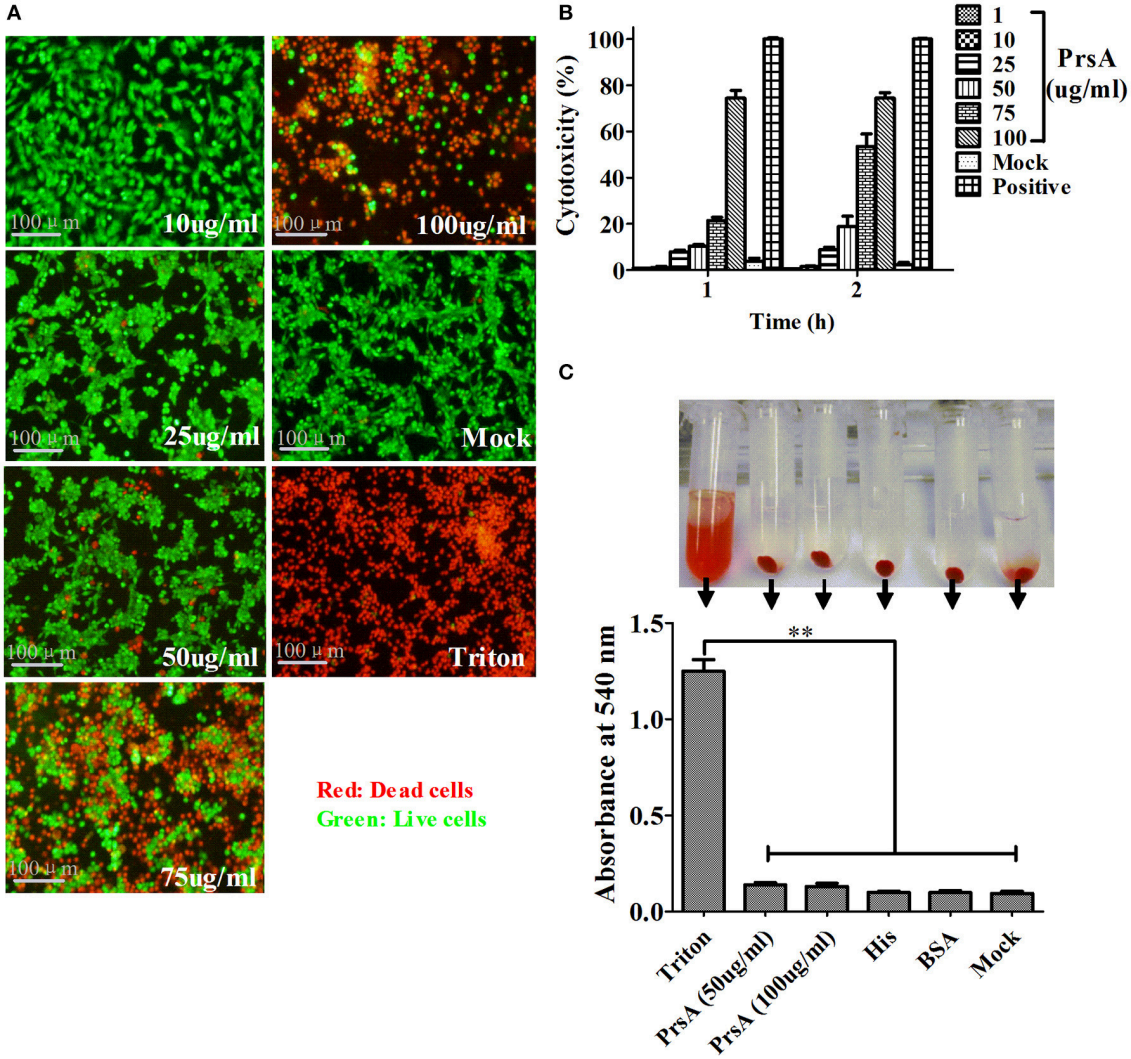
**PrsA protein showed dose-dependent cytotoxicity but without hemolytic activity. (A)** Fluorescent observation of live (green) and dead (red) bEND3.0 cells treated with PrsA at different concentrations for 1 h. Triton X-100 (0.2%) and 1640 medium treatments were used as positive and negative controls. Bar scales indicated were 100 μm. **(B)** Evaluation of cytotoxicity with lactodehydrogenase (LDH) release assay kit. Cells were treated as panel A for 1 h or 2 hs. **(C)** Hemolysis analysis with 2% sheep red blood cells treated with PrsA (50 and 100 μg/ml) for 1 h at 37°C. Triton X-100 (0.2%), His-tag (100 μg/ml) and BSA (100 μg/ml) proteins were used as controls. Absorbance values of the supernatant samples from the above treatments were measured at 540 nm. Data were shown as mean ± SD of three replicate wells of each treatment. ^**^Indicated significant at *P* < 0.01.

**Figure 7:**
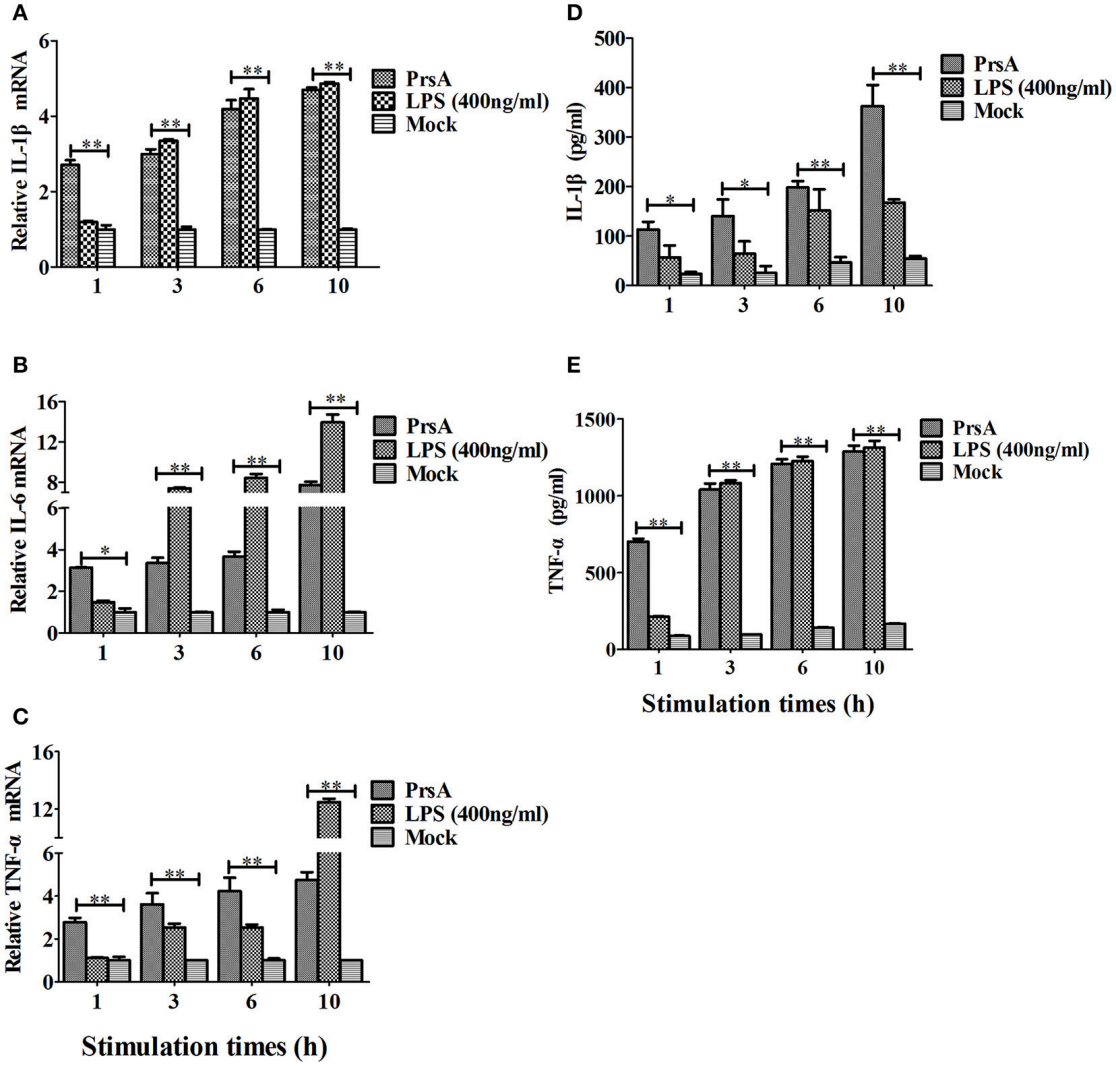
**PrsA protein induced pro-inflammatory cytokine responses in murine macrophage cells. (A–C)** Relative mRNA levels of proinflammatory cytokines IL-1β, IL-6 and TNF-α as determined by qPCR. The results were presented as fold induction relative to non-infected cells against β*-actin* gene expression using 2^−ΔΔCt^ method. RAW264.7 cells were stimulated by PrsA (10 μg/ml) for 1 h, 3 hs, 6 hs, and 10 hs, respectively. Lipopolysaccharide (LPS) was used at 400 ng/ml as controls. **(D,E)** Levels of IL-1β and TNF-α in the culture supernatants of cells treated with PrsA as determined by ELISA. All experiments were repeated three times with similar trends. Data were shown as mean ± SD of three replicate wells of one typical treatment. “^*^” or “^**^” indicated significant at *P* < 0.05 or *P* < 0.01, respectively.

Among the 10 proteins of significant difference, PrsA that is known to be involved in protein secretion and folding was nearly 3 to 9-fold lower in the VirD4 deletion mutant than its parent strain. To explore its functions, the PrsA protein was expressed in soluble form in *E. coli* and purified to homogeneity (Figures 5A,B) with effective removal of endotoxin (less than 0.2 endotoxin units per milliliter). Western blotting and ELISA indicated that deletion of *virD4* reduced the expression of PrsA only upon exposure to hydrogen peroxide (Figures 5C,D). Significant cytotoxicity to bEND3.0 cells was observed with PrsA protein level above 75 μg/ml (Figures 6A,B). However, PrsA did not have hemolytic activity (Figure 6C). RAW264.7 cells stimulated with PrsA at a concentration of 10 μg/ml showed a time-dependent increase of the proinflammatory cytokines as examined by qPCR (IL-1β, IL-6, and TNF-α) (*P* < 0.01, Figures 7A–C) and ELISA (IL-1β and TNF-α) (*P* < 0.01, Figures 7D,E).

## Discussion

Of 35 *S. suis* serotypes, SS2 has emerged as an important zoonotic agent and causes unusual outbreak of streptococcal toxic-shock-like syndrome (STSS) (Gottschalk et al., 2010; Feng et al., 2014). Type IV secretion system (T4SS) is a versatile secretion system important for virulence and even survival of some bacterial species. It was once believed to be present only in Gram-negative bacteria but was recently identified in Gram-positive *S. suis* type 2 strains causing STSS outbreaks in China (Wallden et al., 2010). This putative SS2-T4SS is contained in the 89K pathogenicity island and proposed as a new T4SS subgroup (Type-IVC secretion system) (Zhang et al., 2012). T4SS contains a channel enabling secretion of proteins and DNA molecules across the cell envelope. This translocation is driven by a number of cytoplasmic ATPases that energize conformational changes in the translocation complex. VirD4 is considered as a key coupling protein that could recruit substrates to T4SS for translocation (Wallden et al., 2010; Trokter et al., 2014). In *Helicobacter pylori*, VirD4 presumably acts as an adapter protein guiding CagA into the transport channel and may play a role in inducing host pro-inflammatory responses in both VirD4-CagA-dependent and VirD4-CagA-independent mechanisms (Selbach, 2002). Here we show that VirD4 play important roles in SS2 infection, such as evasion from phagocytosis and increased release of proinflammatory cytokines.

Deletion of the *virD4* gene decreased SS2 virulence in mice, as shown by higher LD_50_ value, lower bacterial load in organs and reduced survival in fresh mouse blood. These virulence phenotypes were similar to the previous findings of another SS2 strain (05ZYH33) with *virD4* deletion (Zhao et al., 2011). However, we found that SS2-VirD4 had a novel antiphagocytic function as evidenced by increased number of bacterial cells in phagocytic cell lines upon *virD4* deletion. In *H. pylori*, putative T4SS core components virB7 and virB11 (ATPase essentially similar to VirD4) were antiphagocytic (Ramarao et al., 2000; Tegtmeyer et al., 2011). Thus, we assume that VirD4 is involved in *S. suis* virulence possibly by evasion from phagocytosis.

Once *S. suis* colonization and invasion of epithelial cells of the respiratory tract and entry to the systemic circulation or deep tissues, SS2 faces phagocytosis by neutrophils and macrophages, the first line of host defense. Phagocytosis-associated respiratory burst would generate antimicrobial reactive oxygen species (ROS) including superoxide anion (${\text{O}}_{2}^{•-}$), hydrogen peroxide (H_2_O_2_), and hydroxyl radical (^•^OH) (Forman and Torres, 2002; Fang et al., 2015; Zheng et al., 2016). We attempted an *in vitro* oxidative stress assay using non-lethal dose of hydrogen peroxide to mimic host-bacteria interaction *in vivo* to examine if such exposure would affect expression of virD4 itself and hence the release of secreted proteins. We did find significantly higher expression of *virD4* mRNA in SS2 not only upon H_2_O_2_ stress but also upon phagocytosis, suggesting that it might be an *in vivo* induced gene. Such oxidative stress also led to differential expression of secreted proteins between WT and ΔVirD4 strains. The protein spots with a >3-fold abundance change due to H_2_O_2_ stress were subjected to MALDI-TOF/MS analysis. Results showed that ten proteins identified are involved in DNA damage repair, nucleotide biosynthesis, carbohydrate metabolism and stress modulation reported in *S. suis* or other bacteria. These secreted proteins with currently unknown mechanisms could be termed as moonlighting proteins that function not only in catalytic or metabolic activities but also act as modulators in bacterial virulence (Henderson and Martin, 2011, 2013).

The PrsA protein secreted abundantly in WT strain, but not in ΔVirD4 mutant, was selected for further evaluation of its functions. Because prokaryotes and eukaryotes are known to have three ubiquitously distributed enzymes known as cyclophilins, FK506 binding proteins (FKBPs), and parvulins, which catalyze the cis/trans isomerization of peptide bonds preceding prolyl residues, thereby assisting protein folding at the post-translocational level (Ünal and Steinert, 2014; Nath and Isakov, 2015). We found that SS2-PrsA expressed in *E. coli* showed dose-dependent cytotoxicity with significant cell death at concentrations above 75 μg/ml. At a non-cytotoxic dose of 10 μg/ml, PrsA induced significant expression of IL-1β and TNF-α in murine macrophage cell line RAW264.7. These results suggest that PrsA may contribute to the pathogenicity of *S. suis* type 2, as its counterparts in other bacterial species that function in a number of biological processes, such as colonization or invasion, protease exportation, immune activation and cells apoptosis (Ünal and Steinert, 2014). In gram-positive bacteria, PrsA is the only general factor mediating folding of secreted proteins essential for bacterial pathogenicity and cell wall biosynthesis (Sarvas et al., 2004). In *S. pneumoniae*, PpmA, a surface-associated homology of the parvulin protein, was demonstrated to contribute to pneumococcal pathogenesis since *PpmA* gene deletion reduced bacterial persistence in mice nasopharynx and enhanced bacterial uptake by macrophages (Cron et al., 2009). In *Listeria monocytogenes*, PrsA2 plays a critical role in folding of virulence factors, cell wall biosynthesis and resistance to osmotic stress (Alonzo and Freitag, 2010; Cahoon and Freitag, 2014). The parvulin peptidyl-prolyl isomerase HP0175 in gram-negative *Helicobacter pylori* induces apoptosis of gastric epithelial cells and modulates the inflammatory response during infection (Basak et al., 2005; Kundu, 2013).

Taken together, we demonstrate that VirD4 is antiphagocytic and its expression increases upon oxidative stress or phagocytosis. PrsA is cytotoxic and proinflammatory, and its expression is dependent on VirD4. We suppose that there might be complex relationship between PrsA and VirD4 and their expression upon oxidative stress during respiratory burst, between VirD4 and phagocytosis, and between PrsA and cytotoxicity in *in vivo* conditions. Further research is required to examine if VirD4 has direct effects as an antiphagocytic protein or results from increased expression of PrsA that exerts cytotoxic effects to debilitate the phagocytic function. The roles of putative SS2-T4SS in PrsA secretion and the possible mechanisms of PrsA in inducing cells death and proinflammatory response are also worth investigation.

## Author contributions

WF and XJ designed the experiments. XJ, YY, and JZ performed the experimental work and data collection. Other authors contributed equally to animal test, literature search, data analysis and interpretation. XJ and WF wrote the manuscript.

### Conflict of interest statement

The authors declare that the research was conducted in the absence of any commercial or financial relationships that could be construed as a potential conflict of interest.
