# Supplementary material for: Roles of the Putative Type IV-like Secretion System Key Component VirD4 and PrsA in Pathogenesis of Streptococcus suis Type 2
Source: Front Cell Infect Microbiol. 2016 Dec 2;6:172. doi: 10.3389/fcimb.2016.00172 (PMC5133265; doi:10.3389/fcimb.2016.00172)
Supplement: Supplementary file 1 [file DataSheet1.doc]

**Supplemental data：**


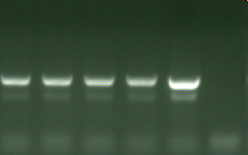

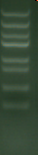

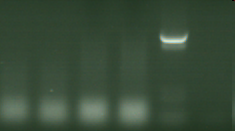


**5k**

**5k**

**VirD4-OF/OR**

**Mock**

**WT**

**△VirD4**

**VirD4-OF/IN2**

**VirD4-IN1/2**


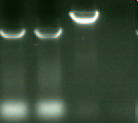

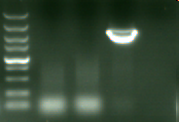

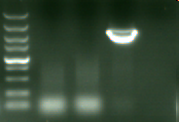

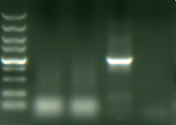

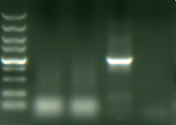


**Mock**

**WT**

**△VirD4**

**Mock**

**WT**

**△VirD4**

**5k**

***cps2J/gdh***

**Mock**

**WT**

**△VirD4**

**SPC-F/R**

**Mock**

**pSET4S**

**WT**

**△VirD4**

**Figure S1. Confirmation of ΔVirD4 mutant strain by PCR.**

The *virD4* deletion mutant strain was verified by PCR and sequencing. Primer pairs VirD4-IN1/VirD4-IN2, VirD4-OF/VirD4-OR, VirD4-OF/VirD4-IN2 and SPC-F/R were used for double crossover identification. The *cps2J/gdh* du-PCR was used for *S. suis* type 2 identification (Table S1). Direct DNA sequencing was applied for verification of the mutation sites using the amplified product with primers of VirD4-OF/OR.

**
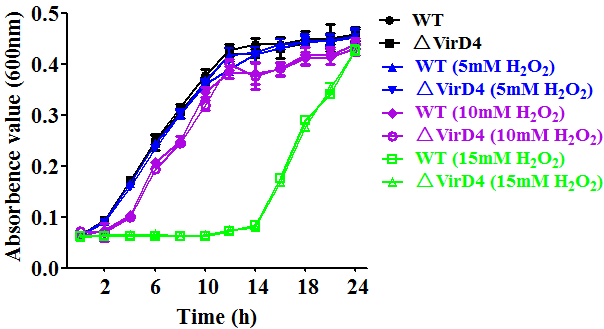
**

**Figure S2. Optimization of hydrogen peroxide concentrations for oxidative stress assay.** The wild-type and △virD4 strains were cultured in BHI with 0, 5, 10 and 15 mM of H_2_O_2_, respectively. Growth under oxidative stress was determined every 2 hours by measuring optical density at 600nm.

**Table S1. Primers used in this study**

| **Primers** | **Sequence (5’-3’)** | **Function** |
| --- | --- | --- |
| VirD4-up-F (*BamH*I) | CGCAGGATCCTCCCATCCTCCTACATCC | VirD4 upstream  fragment amplification |
| VirD4-up-R | TTGTATAGAAATGAGGAATTATAT |
| VirD4-down-F | TTCTATACAAGGTCACCTCCTT | VirD4 downstream fragment amplification |
| VirD4-down-R (*EcoR*I) | AGGTGAATTCAGACAAGATGGGTTGAG |
| VirD4-IN1 | ATAGTGCCACCAGCGTTCA | VirD4 intra-fragment amplification |
| VirD4-IN2 | AAGGGAGACAATCAGGACAAAA |
| VirD4-OF | GTCTGGCACCGTTAGT | outer-regions of VirD4 flank amplification |
| VirD4-OR | AGGTGACAGGGTAGGT |
| SPC-F | GTTCGTGAATACATGTTATA | SPC gene identification |
| SPC-R | GTTTTCTAAAATCTGAT |
| *cps2J*-F | TGAGTCCTTATACACCTGTT | *S. suis* type 2 du-PCR identification |
| *cps2J*-R | AGAAAATTCATATTGTCCACC |
| *gdh*-F | CCATGGACAGATAAAGATGG |
| *gdh*-R | GCAGCGTATTCTGTCAAACG |
| q*VirD4*-F | CAAACCTCAACAGTTCGCTCT | *S. suis* VirD4 mRNA level Primers |
| q*VirD4*-R | CCAAATAGAGGAGGCTGTC |
| q*IL-6*-F | TTCCATCCTGTTGCCTTCTT | qPCR primers for mouse Il-6 |
| q*IL-6*-R | AACTCTTTTCTCATTTCCACGA |
| q*IL-1β*-F1 | CCCAGGGCATGTTAAGGAGCT | qPCR primers for mouse IL-1β |
| q*IL-1β*-R1 | CTTGGCCGAGGACTAAGGAGT |
| q*TNFα*-F1 | CTGAGGTCAATCTGCCCAA | qPCR primers for mouse TNFα |
| q*TNFα*-R1 | GAGCCATAATCCCCTTTCTA |
| q*MCP-1*-F1 | GCAACTCCCATCCCAATCAC | qPCR primers for mouse MCP-1 |
| q*MCP-1*-R1 | CCCTTCTGTCGCCGTCAAA |
| q*β-actin*-F1 | CTCGATCATGAAGTGCGACGT | qPCR primers for mouse β-actin |
| q*β-actin*-R1 | GTGATCTCCTTCTGCATCCTGTC |
| q*IL-1β*-F2 | CAAAAGCCCGTCTTCCTGG | qPCR primers for swine IL-1β |
| q*IL-1β*-R2 | GCTGTTCCTCCTCACTGG |
| qTNFα-F2 | CACGCTCTTCTGCCTACTG | qPCR primers for swine TNFα |
| qTNFα-R2 | GACGGGCTTATCTGAGGTTTG |
| q*IFN-γ*-F | AAGTACCTCAGATGTACCTA | qPCR primers for swine IFN-γ |
| q*IFN-γ*-R | AAGTCATTCAGTTTCCCAG |
| q*MCP-1*-F2 | AAGAGTCACCAGCAGCAA | qPCR primers for swine MCP-1 |
| q*MCP-1*-R2 | TAGGGCAAGTTAGAAGGAA |
| q*β-actin*-F2 | GACCTGACCGACTACCT | qPCR primers for swine β-actin |
| q*β-actin*-R2 | CGTTGCCGATGGTGATG |
| qB13-F | CTTCGGTAGATATGAAGACGT | Differential expressed protein spots’ transcriptional level determination primers |
| qB13-R | CAGCACAGTAGCAACAAATGA |
| qB31-F | TTACAGAAGCAGTTCGTGGC |
| qB31-R | GGCATCATCAAAGATAACATAAGC |
| qB33-F | GTTCTTATGATACTTTGCCACC |
| qB33-R | TTTACCGTCTTTCAATGCTGG |
| qA20-F | CTGGGGTTGTTCCTGTTATG |
| qA20-R | GCGAACTTTCTCATCGGCATTTT |
| qA39-F | GATACACCAGAAACGCTACATTC |
| qA39-R | CCCAACTAGATATTGCCCATC |
| qA120-F | CAAAGGATTTTCGTGAGTTGGTT |
| qA120-R | CTGTTTTAGTGATTGGCGTTTC |
| qA132-F | GTATTGGACTTTGGGACTTATGA |
| qA132-R | GTACCTTGTTTACAGCATTGTTG |
| qA143-F | GGTGATATGGTTGCTGCTGG |
| qA143-R | ATCAAACGAAGCCCTTTGGACTC |
| qA146-F | GGTTTTGCGAAAGAAAGTGG |
| qA146-R | TTGGTATGGCTTCAAGAGGG |
| qA148-F | CCTACTGTGGAGCAGATGGA |
| qA148-R | TAGGCAATCTGGTAATCGACTAG |
| q*16S rRNA*-F | GTAGTCCACGCCGTAAAC | *S. suis 16S rRNA* mRNA level primers |
| q*16S rRNA*-R | TAAACCACATGCTCCACC |
| q*GAPDH*-F | TGGTGGTGACCTTCGTCGTG | *S. suis GAPDH*  mRNA level primers |
| q*GAPDH*-R | CTGGGATTACCAAGCCGATAGC |
| *PrsA*-F (*BamH*I) | CGCGGATCCATGAAACAAACTAAAAAAATTCTCG | PrsA protein expression |
| *PrsA*-R (*Xho*I) | CCGCTCGAGTTACTGACCTGATGAACTAGAA |
